# Chitosan-Based Nanoparticles with Optimized Parameters for Targeted Delivery of a Specific Anticancer Drug—A Comprehensive Review

**DOI:** 10.3390/pharmaceutics15020503

**Published:** 2023-02-02

**Authors:** Joanna Kurczewska

**Affiliations:** Faculty of Chemistry, Adam Mickiewicz University, 61-614 Poznań, Poland; asiaw@amu.edu.pl; Tel.: +48-61-829-1565

**Keywords:** chitosan nanoparticles, targeted drug delivery, anticancer drugs, potential antitumor agents

## Abstract

Chitosan is a positively charged polysaccharide obtained through chitin deacetylation. It belongs to a group of biodegradable, bioavailable, and non-toxic materials of natural origin; thus, it is a promising matrix for creating delivery systems of different active agents. Recently, much attention has been paid to nanodelivery systems as carriers to enable better bioavailability, and thus higher efficiency of the loaded drug. The present review is focused on the progress in chitosan-based nanoparticles for the targeted delivery of antitumor drugs. The paper discusses literature reports from the last three years in which chitosan nanoparticles were applied as carriers for active substances used in antitumor therapy and potential new drugs with anticancer properties. Special attention was paid to the different treatments applied to increase the therapeutic effectiveness and minimize the side effects of a specific active substance.

## 1. Introduction

The needs of the modern world drive the search for new materials applied in various areas of life. Environmental pollution and activities aimed at developing green chemistry stimulate significant interest in materials of natural origin. An undoubted advantage of materials obtained from natural sources is their availability. This can sometimes result in lower cost, compared to those obtained by synthesis. Nevertheless, their practical use is determined mainly by specific parameters that must be met by a given material dedicated to a certain application. Particularly high requirements are placed on biomedical materials. Their biodegradability, biocompatibility, and low toxicity are crucial to finding such applications. A significant group that meets these conditions are biopolymers, among which polysaccharides are one of the most important families [1,2,3].

Polysaccharides, composed of different monosaccharides combined by glycosidic bonds, are characterized by several reactive functional groups capable of chemical modification. Their unique properties are often used in drug delivery systems’ (DDSs) design. DDSs are an alternative to traditional forms of drug administration. They are designed to reduce the toxicity of the active substance and its side effects and increase the drug’s effectiveness by protecting the active substance delivered to the target environment. This type of solution is particularly desirable for drugs used in cancer therapy.

Based on The International Agency for Research on Cancer (IARC) and Global Cancer Data (GLOBOCAN 2020), nearly 10 million deaths caused by cancer were registered in 2020 (over 4 million in Asia and about 1 million in Europe). Global cancer mortality is often analyzed using age-standardized rate, ASR (per 100,000 population). The highest cancer mortality for both sexes was in Mongolia (175.9), while in separate sexes—it was in Zimbabwe (142.9 women) and Mongolia (224.3 men).The most significant number of new cases (out of all 18.1 million) concerned breast (the most common in women) and lung (the most common in men) cancer (over 2 million each), colon and rectum, prostate, skin, and stomach cancer (over 1 million each) [4]. Treatment procedures include three elements, i.e., surgery, radiotherapy, and systemic therapy (including chemotherapy, hormonal treatment, and targeted biological therapies). Chemotherapy involves the long-term use of highly toxic active substances, e.g., doxorubicin or paclitaxel. The number of literature reports on new potential DDSs for anticancer drugs indicates that effective materials dedicated to such applications include various groups of compounds. Nevertheless, polysaccharide biopolymers’ well-known and desirable properties make them particularly useful in this application. One of the commonly used biopolymers as the basis for drug carriers, including anticancer drugs, is chitosan (CS).

### 1.1. Structure and Properties of Chitosan

**Figure 1:**
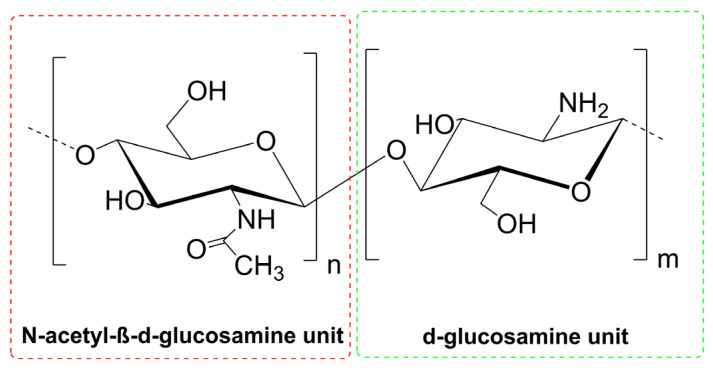
The structure of chitin/chitosan composed of N-acetylglucosamine and D-glucosamine units: m < n chitin, m > n chitosan.

Chitosan represents a linear polysaccharide that is obtained through the deacetylation of chitin. The significant presence of chitin, composed of acetylglucosamine units, in many organisms—crustaceans, algae, mollusks, insects, or fungi cell walls—makes it one of the most abundant and widespread biomolecules in the world [5,6]. Deacetylation of chitin by chemical (sodium hydroxide solution) or enzymatic treatment involves removing acetyl groups and substituting amine functional groups. The degree of deacetylation (DD), defined as a percentage of glucosamine monomers in the polysaccharide structure, is the limit value used to distinguish the structure of chitin and chitosan. The structures with a DD value above 50% are defined as chitosan; therefore, it is a copolymer of N-acetylglucosamine and D-glucosamine units (Figure 1). Commercially available chitosan usually has a DD of 75–90%. Based on DD value, chitosan can be categorized as low (55–70%), middle (70–85%), high (85–95%), and ultrahigh deacetylated (95–100%). In general, deacetylation never reaches 100% [7]. DD and molecular weight strongly influence chitosan properties and thus its potential application.

The presence of amino groups imparts a cationic character to the polysaccharide and affects its solubility. Due to amine groups’ protonation, this weak base is only soluble in dilute acidic solutions and insoluble in water and organic solvents. The higher the number of amine groups, the lower the molecular weight and the better the solubility. Chitosan can be categorized as low (below 90 kDa), medium (190–310 kDa), and high (310–375 kDa) molecular weight CS. As the molecular weight increases, the viscosity of the polysaccharide is also higher. Importantly, chitosan with a molecular weight below 30 kDa is easily soluble in water without acid addition, while polysaccharides with a molecular weight above 30 kDa require an acidic environment for the protonation of amino groups. The solubility decreases as the molecular weight increases due to the increasing influence of hydrogen interactions between the chains [6]. Additionally, reactive amine and hydroxyl functional groups make this biopolymer susceptible to chemical modifications to improve or obtain new properties.

**Figure 2:**
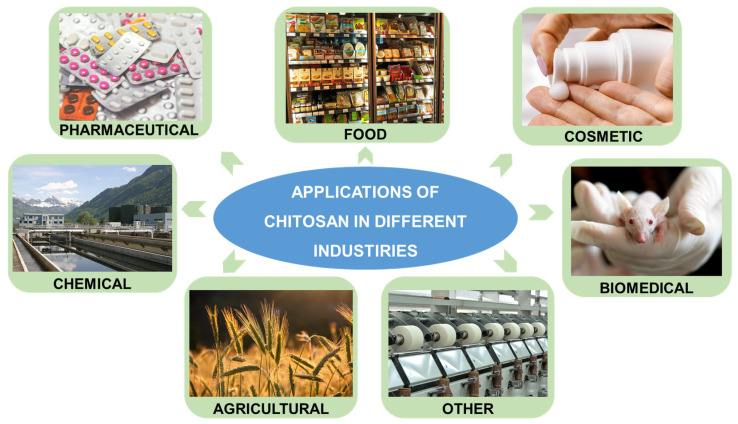
A schematic representation of diverse applications of chitosan-based materials.

The uniqueness of chitosan results from its numerous valuable properties, which enable its application in many branches of industry (Figure 2). This low-cost material is characterized by biodegradability, biocompatibility, and low toxicity; thus, it is a safe product that can be successfully used in the pharmaceutical, biomedical, food, or cosmetic industries. The biological properties are also essential, i.e., antifungal, anti-inflammatory, antimicrobial, antioxidant, antitumoral, or mucoadhesive activities [8]. They can be successfully used for therapeutic purposes and in other industries, such as food, agriculture, or other areas (e.g., in the textile industry). In addition, the high adsorption capacity of chitosan is used for environmental applications to remove harmful impurities from aqueous solutions.

### 1.2. Drug Delivery Systems Based on Chitosan

The number of potential biomedical and pharmaceutical applications of chitosan-based materials is impressive and constantly expanding. The most critical areas where chitosan is used are drug delivery, cancer therapy (including delivery of chemotherapeutics), and wound healing combined with the local delivery of active substances [9]. Recently, DDSs based on nanotechnology have been intensively developed as they perform better biological distribution and have higher efficiency than traditional delivery systems [10]. Chitosan-based nanomaterials belong to a large group of nanocarriers represented by liposomes, micelles, nanoemulsions, nanotubes, dendrimers, or polymeric nanoparticles [11,12]. CS nanocarriers, such as nanogels, nanofibers, liposomes, and nanoparticles, are dedicated to varied delivery routes—oral, ocular, pulmonary, transdermal, nasal, vaginal, or parenteral. CS-hydrogels in the nanoscale are responsible for better flexibility and stability of the carrier. The nanogels can be formed without a crosslinker, but unmodified material is only capable of entrapment of hydrophilic drugs. On the other hand, CS-nanofibres allow controlled drug release and improve drug solubility. However, the typical electrospinning method of nanofibers formation is a significant challenge for chitosan due to the high viscosity of its solutions at low concentrations. In the case of liposomes as nanocarriers, the number of advantages is also significant—such as the ability to carry both hydrophilic and hydrophobic drugs, protection of active substances against degradation, reduction in its toxicity, or improvement of penetration. Chitosan in this type of nanocarrier acts as a coating that provides better stability and rigidity of liposomes. However, a high concentration of chitosan results in its aggregation, which in turn destabilizes the nanocarrier. Additionally, the coating based on unmodified chitosan has limited solubility at physiological pH, preventing the active substance’s release [13].

**Figure 3:**
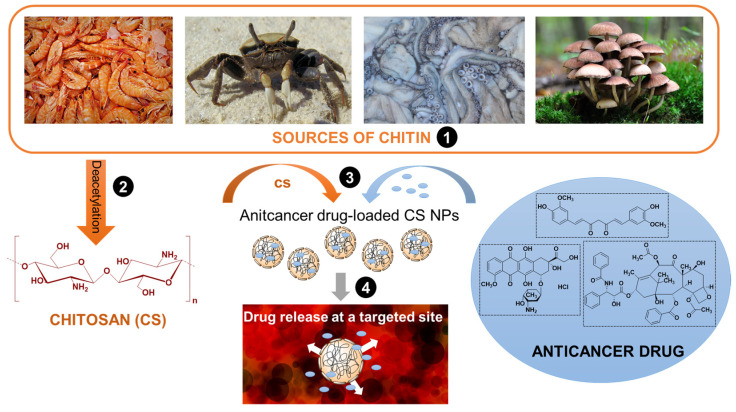
A schematic pathway from natural products to efficient delivery and release of anti-cancer drugs in targeted sites by chitosan-based nanoparticles.

Nanoparticles (NPs) characterized by size below 100 nm are capable of passing blood capillaries and thus efficiently deliver active components to their targeted destination. They can be effectively used for the entrapment of different therapeutic species and allow their controlled release in a differentiated targeted area. Significantly, they contribute to increased circulation, stability, and solubility of active agents. Their ability to cross cell barriers results in increased drug concentration inside desired cells. Chitosan (CS NPs) nanoparticles have several beneficial properties distinguishing them from other nanoparticles, i.e., facility of surface modification, the simple procedure of preparation, high structural stability, excellent solubility, and very good responsiveness to different stimuli [8]. CS NPs can be obtained using different techniques such as ionotropic gelation, the reverse micellar method, emulsion-droplet coalescence, precipitation, or spray drying [13,14,15]. The interest in using chitosan nanoparticles in the delivery of anticancer drugs, results from the properties of chitosan combined with the effect of nanocarriers. The anticancer drugs are mostly loaded into CS NPs using encapsulation technology, but their direct attachment is also used [16,17]. The bioactive molecules are sometimes attached to chitosan through a spacer sensitive to specific environmental conditions, thus enabling a therapeutic effect in the desired area [15]. The anticancer activity of the polysaccharide [18] should enhance the therapeutic effect in combination with classic chemotherapeutics and simultaneously limit their significant side effects. Finally, through the appropriate use of natural resources, an effective and safe nanomaterial for the targeted delivery and release of chemotherapy drugs can be obtained (Figure 3).

Due to the considerable potential of chitosan nanoparticles in cancer therapy, there are numerous studies in this field [19,20], which are often focused on one type of cancer, such as breast [16,21,22], lung [23], or colorectal cancer [24]. However, new original articles are constantly being published, showing the continuing interest, and thus, the still not exhausted potential of the discussed materials. Analyzing the data from the Scopus database for the last three years (2020–2022), it can be seen that the number of original papers on the subject of chitosan nanoparticles is at a level of about two thousand per year, of which more than 500 relate to drug delivery (Table 1). Importantly, a significant part of them included applications related to cancer therapy.

Despite numerous reviews about CS NPs in cancer treatment, there is no detailed comparative analysis of different treatments used to obtain effective nanocarriers for the same anticancer drug. Therefore, the present review is focused on the parameter assessment of a series of nanocarriers based on chitosan nanoparticles in terms of optimizing their parameters for a specific anticancer drug. The author has concentrated on research studies from the last three years (2020–2022) and selected compounds with anti-cancer properties most often used in original articles.

## 2. CS NP Platforms for a Specific Drug Delivery

Table 1 includes information on the number of original papers concerning the use of CS NPs dedicated to specific active agents in anticancer therapy, among which the most significant number of reports concerns doxorubicin [25] and curcumin [26]. Anticancer drugs are divided into two main groups—cytotoxic drugs (acting on cells) and those affecting the hormonal environment. Cytotoxic drugs are categorized into several subgroups, such as antibiotics (e.g., doxorubicin), antimetabolites (e.g., 5-fluorouracil), alkylating agents (e.g., carmustine), platinum complexes (e.g., cisplatin), microtubule damaging agents including vinca alkaloids (e.g., vinblastine) and taxanes (e.g., paclitaxel), camptothecin analogous (e.g., topotecan) and others (e.g., imatinib). Drugs affecting the hormonal environment could be categorized into two main subgroups—steroid hormones (e.g., estrogens) and their antagonists (e.g., tamoxifen). Some commercially available drugs are obtained from products of natural origin (e.g., antibiotics, taxanes, and vinca alkaloids). Nevertheless, intensive scientific research is conducted on numerous compounds of natural origin that have anti-cancer properties but have not yet been approved for chemotherapy (e.g., curcumin). Therefore, this review concerns examples of drugs available on the pharmaceutical market and those with high anticancer potential.

### 2.1. Doxorubicin as a Representative of Antibiotics

**Figure 4:**
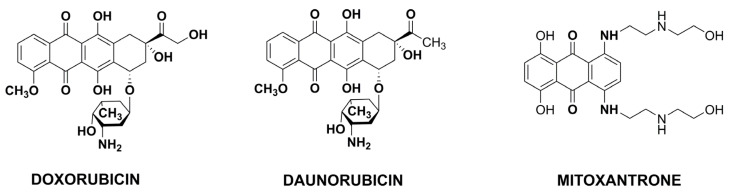
The selected structures of anticancer antibiotics.

Antibiotics are produced by different microorganisms (like bacteria or fungi) but also higher animals and plants. Antitumor antibiotics (Figure 4) are chemicals capable of cancer apoptosis promotion, cancer growth inhibition or cancer metastasis prevention and are used to treat different cancers. They act as nonspecific DNA intercalating agents. The most common classes of anticancer antibiotics are anthracyclines, mitomycin, bleomycin, actinomycin, guanorycin and endiyne [27]. Doxorubicin (Adriamycin), DOX, belongs to the anthracycline antibiotics, characterized by three significant biochemical effects: electron-donating/accepting, production of free radicals, and interaction with cell membranes to form lipid peroxides [28]. DOX belongs to the most effective antitumor agents; thus, it is often used in treating different malignancies. On the other hand, its cardiotoxicity is a significant problem, especially in treating patients with cardiac ailments. Additionally, lack of selectivity and short blood circulation time results in an intensive search for systems that minimize undesirable effects.

The longer blood circulation time of DOX-loaded CS NPs could be achieved by simple coating with polyethylene glycol (PEG). PEGylated chitosan nanoparticles were proposed for targeted DOX delivery [29]. The nanoparticles were fabricated by ionotropic gelation with tripolyphosphate (TPP) as a biologically safe and non-toxic cross-linking agent. After DOX encapsulation within the PEGylated nanosystem, the surface was functionalized to conjugate monoclonal antibodies—anti-human epidermal growth factor or anti-human mammaglobin. The presence of selected monoclonal antibodies should ensure higher selectivity of the doxorubicin drug carrier. The in vitro release studies indicated good stability of the nanoparticles in the physiological environment (pH 7.4), while in cancerous acidic conditions (pH 6.6), initial burst release was followed by a slower controlled one of subsequent DOX doses. The difference in pH values between tumors and normal tissues primarily results from a high metabolism rate, high glycolysis rate, and reduced use of oxidative phosphorylation in tumor cells [8]. Additionally, the presence of breast tumor-specific monoclonal antibodies resulted in high specificity of the nanocarriers, and their much higher cytotoxicity towards breast cancer (MCF-7) cells than free doxorubicin, with a lower response from normal mouse fibroblast cell lines (L-929). On the other hand, glycol-chitosan nanoparticles, similarly to other drug-loaded NPs, could have limited delivery efficacy due to extracellular matrix (ECM) in tumor tissues that inhibits drug carrier penetration. The dense extracellular matrix can be destroyed using high-intensity focused ultrasound (HIFU) technology that should result in enhanced cellular uptake [30]. The therapeutic efficacy of these DOX-loaded NPs was investigated by their injection into A549 tumor-bearing mice. It was found that free DOX was not enough to kill tumor cells, and the chitosan nanoparticles without HIFU treatment demonstrated a similar therapeutic effect. Only after HIFU treatment could the NPs easily penetrate and accumulate in tumor tissues resulting in improved antitumor efficiency.

**Figure 5:**
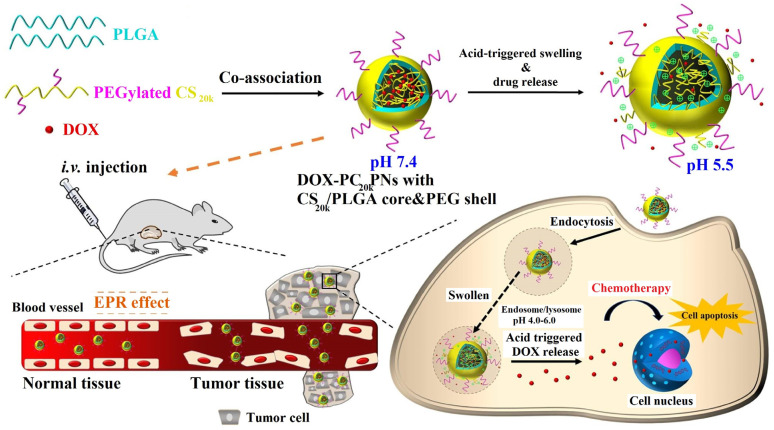
Schematic representation of pH-responsive hybrid nanoparticles as an effective platform for DOX delivery in cancer treatment. Reprinted from [32], Copyright (2022), with permission from Elsevier.

Higher specificity of anticancer drug nanocarrier could also be achieved by designing pH-sensitive devices. An association of pH-responsive, amino-acid-based surfactant to CS nanoparticles with encapsulated DOX, augmented DOX cytotoxicity by improving its antitumor activity in mildly acidic conditions [31]. Another approach to obtain a pH-responsive vehicle was based on the co-assembly of amphiphilic PEGylated chitosan and hydrophobic poly(lactic-co-glycolic acid) (PLGA) units [32]. DOX was encapsulated in a CS/PLGA core covered with a hydrophilic PEG shell (Figure 5). The system studied demonstrated limited DOX release at pH 7.4, but much higher at pH 5.5. Under weak acidic conditions, CS was protonated, resulting in a significant swelling of the nanocarrier. Additionally, the repulsive forces between protonated CS and DOX molecules increased in an acidic environment. On the other hand, non-pH sensitive PLGA nanoparticles demonstrated similar release profiles regardless of pH. The nanocarrier had high colloidal stability provided by the steric repulsion property of the PEG shell and the ability of DOX liberation after acidic activation. Therefore, after their endocytosis in murine prostate cancer cells (TRAMP-C1), DOX was rapidly released (pH 4–6), facilitating the drug accumulation in cancerous cells and thus promoting cell apoptosis. Moreover, the analogous cell destructive effect was not observed for normal (healthy) tissue.

Another approach to obtaining a stimuli-responsive nanoplatform was designed for the co-delivery of doxorubicin and the P-glycoprotein (P-gp) inhibitor (siMDR1) in drug-resistant breast cancer treatment [33]. Unlike the previously discussed examples, DOX was linked to carboxymethyl chitosan through a disulfide bond and formed a negatively charged redox-responsive core of the nanocarrier. The positively charged pH-responsive shell contained siMDR1 to suppress P-gp expression in cancerous cells encapsulated in oligoethylenimine directly. Finally, the nanoplatform surface was modified with AS1411 aptamer and GALA peptide-functionalized hyaluronic acid (AHA/GHA). After endocytosis by tumor cells, carboxymethyl CS was protonated, and the attractive interaction between core and shell was weakened; thus, siMDR1 and the core could be released from the nanoplatform. DOX release occurred later due to cleavage of the disulfide bond in high glutathione concentrations in the cytoplasm. Non-simultaneous release of active ingredients stimulated by redox and pH factors, as well as improved accumulation in tumor and easier transfer of reactive components to cytoplasm due to the presence of hyaluronic acid derivatives, resulted in enhanced anticancer efficiency of the composite nanomaterial for drug-resistant breast cancer. Thus, the nanocarrier demonstrated a tendency for accumulation in cancer cells due to enhanced permeability and retention (EPR) effect and active targeting ability. EPR was also successfully adopted in hybrid nanoparticles based on ortho ester-modified Pluronic cross-linked with chitosan, characterized by high circulation stability and tumor accumulation [34]. Pluronic is an amphiphilic copolymer composed of hydrophilic and hydrophobic blocks, and it is capable of suppressing the abilities of P-glycoproteins and multidrug resistance proteins responsible for significant reduction in cytotoxic drug concentration in tumor cells. The presence of ortho-ester modification resulted in its degradation in mildly acidic conditions; thus, the nanomaterial is pH sensitive, and antitumor activity is significantly increased. Different chitosan modifications could also lead to the desired selectivity, including the formation of cationic amphiphilic NPs [35]. Therefore, chitosan was modified with deoxycholic acid and glycidyl trimethylammonium chloride to obtain soluble micelle-like NPs with positive charge density. 

Another solution for preparing pH-sensitive DDS was based on a prepolymer composed of chitosan oligosaccharide and doxorubicin combined through benzaldehyde-terminated polyethylene glycol by imine bonds [36]. A significant response to pH with the fastest DOX release in a mildly acidic solution was also achieved for the nanoparticles obtained by ionic gelation, using positively charged 2-hydroxypropyltrimethyl ammonium chloride CS with/without grafted folic acid (FA) and negatively charged carboxymethyl CS [37]. The antitumor activities against four different cancer cells were higher than that of free DOX, and the presence of folic acid increased the anticancer activity of the nanoparticles. CS and folic acid were also used to obtain magnetic hybrid NPs for DOX delivery to osteosarcoma cells [38]. Chitosan was modified with succinic anhydride, and folic acid was conjugated through an amide bond. Then CS-FA formed an inclusion complex with magnetite (Fe_3_O_4_). Magnetic iron(II, III) oxide nanoparticles are considered effective materials for targeted drug delivery due to their non-toxicity, small particle size, and ability to be controlled by an external magnet. However, their disadvantage is the tendency to aggregate, which would prevent their penetration and drug delivery to bone tissue where osteosarcoma is present. Thus, the chitosan coating should protect the magnetic core containing DOX, while the presence of folic acid ensures targeted delivery to tumor cells where folic receptors are over-expressed. 

Local chemotherapeutic delivery to cancer cells could also improve the precise delivery of anticancer drugs. Preparation of mucoadhesive nanocarrier should promote local delivery of the cytotoxic drug to the oral cancer cells. Therefore, catechol-modified chitosan NPs were used for the local administration of doxorubicin [39]. Catechol facilitated the carrier adhesion to oral mucosa and enabled sustained DOX release in a targeted oral cavity, which should contribute to reducing systemic side effects. A more sophisticated system for improved DOX targeted delivery included benzylguanidine characterized by high affinity for C-X-C chemokine receptor type 4 (CXCR 4) expressed on the surface of different tumor cells [40]. Benzylguanidine, a guanidinium derivative with a phenyl group, is characterized by similar cationic and amphiphilic properties as cell-penetrating peptides responsible for improved targeted drug delivery. On the other hand, it causes high protein adsorption; thus, the nanoparticles were additionally decorated with galactose bearing lactobionic acid. The composite reduction-responsive nanosystem was stable under physiological conditions, and exhibited DOX release under high glutathione concentration in cancer cells. High affinity interaction of benzylguanidine with CXCR 4 resulted in stronger antitumor efficiency in hepatocarcinoma and breast cancer, and the presence of galactose improved the DOX antitumor effect against hepatocarcinoma. At the same time, lactobionic acid prevented nonspecific protein adsorption, which resulted in better accumulation of the NPs in tumor tissues.

The antitumor activity of doxorubicin could be enhanced by combining different therapeutic agents in anticancer therapy. Combining gene- and chemo-therapies is one of the strategies that should result in a synergistic effect. A method that inhibits target genes is a small interfering RNA (siRNA) technique that leads to silencing cancer gene expression. Simultaneous delivery of DOX and siRNA should increase anticancer therapy effectiveness [41]. An interesting approach for co-delivery of siRNA and DOX was based on chitosan lactate NPs functionalized with a transactivating transcriptional activator (HIV-1 derived TAT peptide) and hyaluronate [42]. Effective siRNA delivery requires a stable drug delivery carrier as the material is unstable, and its transfer to tumor cells is limited. The modified-chitosan nanoparticles were used to encapsulate anti-CD73 siRNA because this cell surface enzyme is highly expressed in tumors. CD73 has a significant impact on the growth and spread of tumor cells. It also contributes to drug resistance; thus, its targeted blockade could be considered a therapeutic method. On the other hand, the presence of cell-penetrating peptides—HIV-1 derived TAT peptide—should result in increased cell uptake of NPs into tumor cells, while hyaluronate coating should contribute to interactions with other molecules overexpressed in cancer cells (CD44). Finally, the nanocarrier should enable improved DOX delivery to tumor cells and reduce effective drug doses. Indeed, a combined therapy resulted in increased cell death and reduced tumor growth compared to free DOX and the drug encapsulated in unmodified CS NPs. Another proposal to combine gene therapy with chemotherapy was based on the co-delivery of DOX and CRISPR/Cas9 or RNAi-expressing plasmid [43]. The clustered regularly interspaced short palindromic repeats (CRISPR) with associated protein 9 (Cas9) gene editing technology and RNA interference (RNAi) were used to inhibit the expression of a selected protein overexpressed in tumors—survivin. The nanoparticles were composed of hydrophilic trimethyl chitosan with a positive charge grafted with folic acid and 2-(diisopropylamine) ethyl methacrylate (DPA). The nanocarrier was pH sensitive due to protonation of DPA primary amine groups in the mildly acidic tumor environment, while the presence of FA resulted in increased NP uptake in tumor cells characterized by a high expression of folic acid receptors. Importantly, antitumor efficiency was higher than those of a single delivery of anticancer therapeutic agents.

Another broadly researched strategy for enhanced therapeutic effects in cancer therapy is using several drugs encapsulated in a nanocarrier. The combination of DOX and raloxifene properties was proposed as a suitable strategy for preparing a targeted platform for breast cancer cells [44]. Raloxifene belongs to selective estrogen receptor modulators that could inhibit human mammary tumor cells. On the other hand, a completely different solution was proposed for estrogen-responsive breast cancer. Estrone was used as a targeting agent and conjugated with doxorubicin and chitosan to obtain a dual estrogen-targeting platform at the nuclear and cellular levels [45]. Other nanocarriers dedicated to breast cancer therapy included DOX and vincristine (VIN). VIN has limited use due to its drastic side effects, including neurotoxicity. Therefore two selected chemotherapeutics were entrapped into niosomes and coated with chitosan to obtain a suitable platform for treating aggressive breast cancer [46]. The pH-responsive nanoformulation increased apoptosis, cell uptake, and endocytosis in breast cancer cells. The metastasis and migration of cancer cells were significantly inhibited compared to a free single drug, and free of both chemotherapeutic drug effects. Another approach to potential use in breast cancer therapy was based on the gold/chitosan nanocomposites loaded with two anticancer drugs—doxorubicin and 5-fluorouracil [47]. On the other hand, an interesting proposal dedicated to non-small lung cancer was the use of glycol-CS nanoparticles conjugated to DOX, co-loaded with nonsteroidal anti-inflammatory drug celecoxib, and finally covered with hyaluronic acid [48]. Hyaluronic acid has a strong binding affinity to CD44 receptors overexpressed in tumor cells. DOX was conjugated by a pH-sensitive linker, ensuring bonding stability in a physiological environment and drug release in acidic conditions specific for tumor cells, while the combination of cytotoxic and anti-inflammatory drugs resulted in a synergistic effect.

Many literature reports have recently used doxorubicin with compounds of natural origin to obtain synergistic effects in cancer therapy. Such a nanocarrier has been proposed using a hydrophobic oleanolic acid widespread in plants, grafted onto hydrophilic chitosan oligosaccharide [49]. The chitosan-based core was covered with a hyaluronic acid derivative shell bearing doxorubicin attached through an amide bond. The two reactive components of the nanomaterial were activated in the tumor environment, and antitumor efficacy was improved compared to mono-delivered drugs. A different approach to delivering two therapeutic agents was proposed using DOX and hydrophobic curcumin with high anti-cancer potential [50]. The redox-responsive nanocarrier was prepared using thiolated chitosan and thiolated stearic acid to obtain disulfide cross-linked nanoparticles without the addition of cross-linking agent. The nanoparticles could load hydrophobic and hydrophilic reactive agents and demonstrated high stability in physiological pH while being degraded in significant glutathione concentrations. It was proven that glutathione is produced in many cancerous cells at a much higher concentration (2–20 mM in the cancer cell cytoplasm), than in healthy cells (2–20 μM in the extracellular matrix and body fluids of normal tissues) [51,52]. It is responsible for disulfide bond destruction resulting in anticancer drug release, and it is usually applied as a redox-responsive agent in delivery systems targeted to cancer cells [8]. Additionally, the drugs encapsulated in the NPs demonstrated higher cancer cell efficiency than each free drug. The disulfide bond was also adopted in other CS-based nanoparticles for the co-delivery of doxorubicin and quercetin [53]. Initially, trimethyl chitosan was modified with PEG, and then DOX was conjugated via a disulfide bond to obtain a redox-sensitive medium. Finally, quercetin was encapsulated into such nanoparticles. The nanocarrier demonstrated enhanced drug release ability in high glutathione concentrations and increased tumor inhibition efficacy. On the other hand, a species of natural origin with potential antitumor activity could also be chemically bonded to chitosan and then used for loading cytotoxic DOX. For this purpose, CS was functionalized with hydrophobic cinnamaldehyde (CA) to obtain new biological properties and hydrophilic dimethyl maleic anhydride to give the material the ability to react with amino groups and form of β-carboxylic amides [54]. The amide bond was labile in an acidic tumor environment, resulting in higher cellular uptake. Further pH decrease resulted in DOX and CA release, which manifested an increased toxicity in breast cancer cells. An interesting synergistic antitumor effect was studied for lactobionic acid (LA) and CA-modified chitosan with encapsulated DOX [55]. LA modification improved NPs uptake by tumor cells, DOC and CA were released in tumor cells due to depolymerization, and CA stimulated cells, to produce reactive oxygen species that increased the oxidative stress of cancer cells and induced their apoptosis. At the same time, DOX had a cytotoxic effect directly on tumor cells.

Other proposed solutions assume a combination of chemo- and immunotherapy. Co-delivery of DOX and interleukin-12, a proinflammatory chemokine potentially responsible for cancer cell elimination and inhibiting cancer angiogenesis, in nanoparticles composed of cationic chitosan and anionic poly-(glutamic acid) contributed to better stability of NPs in blood, co-release of anticancer agents in the tumor environment and a high rate of tumor inhibition [56].

The above-described examples of the latest literature reports suggest that effective targeted DOX delivery in chitosan-based nanoparticles could be achieved by using simple drug encapsulation in CS NPs, other nanoparticles’ coating, incorporation of other components for improved targeting to cancer cells or chemical DOX bonding sensitive to redox or pH conditions. Table 2 includes selected chitosan-based nanocarriers used for targeted DOX delivery characterized by various solutions in the construction of carriers for better-targeted delivery of the antibiotic, reducing its toxicity to healthy cells, reducing the therapeutic dose or directing its action to a specific type of cancer cells.

### 2.2. Antimetabolites

**Figure 6:**
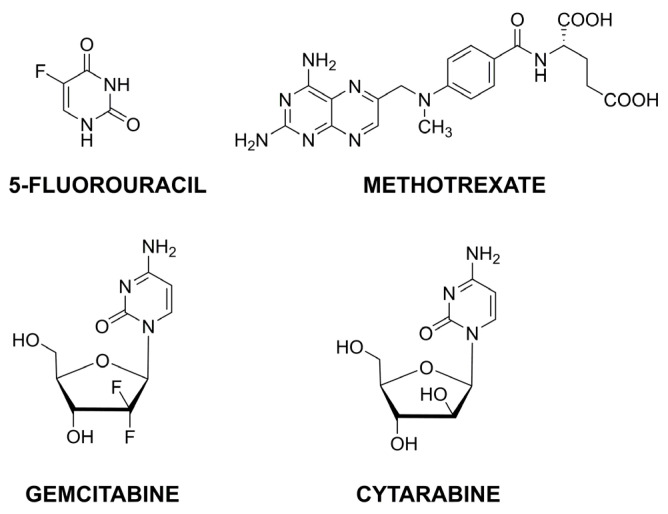
Selected structures of anticancer antimetabolites.

Another critical group of anticancer drugs is the antimetabolites, responsible for interfering with DNA/RNA formation and substituting their regular building blocks. Examples of the structures of this group of compounds are presented in Figure 6.

#### 2.2.1. 5-Fluorouracil

A major antimetabolite drug is 5-fluorouracil (5-FU), a pyrimidine analog responsible for inhibiting thymidylate synthase. However, its rapid metabolism, high toxicity, and drug resistance, stimulate research studies into improved delivery systems. A composite of chitosan/polycaprolactone (CS/PCL) nanoparticles was applied for 5-FU entrapment to obtain higher neoplastic activity in head and neck cancer [57]. On the other hand, different folate-modified CS nanoparticles were proposed for targeted drug delivery to colon cancer cells. For that purpose, CS was modified with FA to enhance NPs accumulation in tumor cells, characterized by the presence of folate receptors, and then encapsulated in alginate beads [58]. Calcium alginate should protect the entrapped drug against its release in the gastric environment and impart mucoadhesive properties to the nanocarrier for prolonged intestinal residence time. A slightly different solution for a nanocarrier with an analogous application was based on the use of folate and lipid modification of chitosan [59]. A similar assumption was made for the 5-FU nanocarrier dedicated to targeting liver cancer. Biotin (Bio), a water-soluble vitamin, can be used as another targeting tumor ligand as Bio receptors are overexpressed on the surface of tumor cells. Therefore, another effective nanosystem for 5-FU delivery was based on dual cancer-cell targeting agents—FA and Bio [60], characterized by improved targeting to liver cancer cells, sustained 5-FU release in the desired environment, and reduced toxic side effects.

Another direction of research on optimal 5-FU carriers is related to the use of inorganic compounds. Cerium oxide nanoparticles demonstrate the scavenging ability of reactive oxygen species (ROS) and thus can be used in preparing ROS-responsive drug delivery systems. For that purpose, 5-FU-loaded CS NPs were coated with cerium oxide [61]. CeO_2_ resulted in improved scavenging ability, while pH sensitivity resulted in improved apoptosis in hepatocellular carcinoma cells. On the other hand, a more complex nanosystem, including gamma-alumina (γ Al) and magnetite (Fe_3_O_4_), was used as a potential 5-FU carrier dedicated to breast cancer treatment [62]. Aluminum oxide possesses several beneficial properties for drug delivery—high surface area and stability, including its dispersion in water. Iron oxide nanoparticles are applicable for biomedical purposes due to their non-toxicity, tissue penetrating ability, or superparamagnetism. The synthesized alumina nanopowder was introduced into a chitosan solution, followed by adding magnetite nanoparticles and 5-FU loading. The uniform hydrogel was treated with surfactant, dropwise to oil, and finally treated with polyvinyl alcohol to obtain 5-FU loaded CS/γ Al/Fe_3_O_4_ composite nanoparticles. The presence of iron oxide contributed to improved drug loading. The nanosystem demonstrated good stability and pH-sensitive drug release in the tumor environment.

Additionally, some literature reports include combined targeting of different reactive agents to obtain synergistic antitumor activity. Based on this assumption, nonsteroidal anti-inflammatory aspirin was co-loaded with 5-FU in chitosan NPs [63]. Aspirin contributed to reduced cell viability and thus strengthened the antitumor effect of 5-FU. A more sophisticated nanosystem was designed to combine chemo- and photothermal therapy. The latter is based on noninvasive ablation to tumorigenic cells using near-infrared light. It should also facilitate cytotoxic drug penetration into cell membranes. Polypyrrole belongs to light-absorbing molecules with good biocompatibility. Therefore, it can produce a photothermal conversion effect. For this purpose, polypyrrole and 5-FU chitosan NPs were covered with carboxymethyl cellulose cross-linked with disulfide to obtain a multi-responsive nanocarrier with the synergistic effect of two antitumor therapy agents [64]. The complex nanoparticles displayed high response selectivity under near-infrared irradiation, high glutathione concentration, and a weakly acidic environment. 

#### 2.2.2. Other Antimetabolites

Other antimetabolites are less frequently found in literature reports, but some interesting nanocarriers are also described. Methotrexate (MTX) is an analog of folic acid responsible for blocking dihydrofolate reductase and thus inhibiting FA metabolism. However, its low oral bioavailability and permeability limit drug application in systemic therapy. To overcome these disadvantages, composite nanoparticles prepared by self-assembling cationic chitosan and heparinoid anionic sulfated polysaccharide—fucoidan-were synthesized [65]. The NPs were dedicated to oral MTX delivery in lung cancer therapy. The nanocarrier demonstrated good mucoadhesive properties, desirable for oral drug administration. Moreover, the anticancer properties of fucoidan resulted in a synergistic effect. Therefore, a therapeutic dose of MTX could be reduced. 

**Figure 7:**
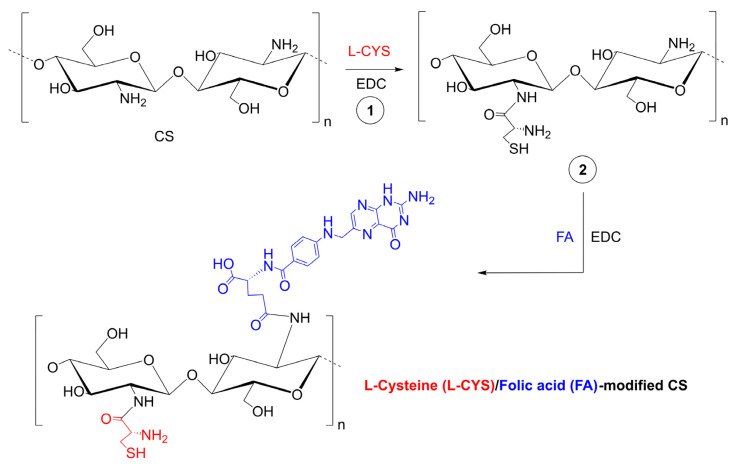
Synthesis of folic-thiolated chitosan in the presence of 1-ethyl-3-(3-dimethylamino propyl) carbodiimide (EDC). Adapted from [67], Pharmaceutics 2020.

Another strategy for MTX high loading and efficient delivery used two types of interactions—electrostatic and multiple hydrogen bonding [66]. Chitosan oligosaccharide with amine groups interact electrostatically with carboxyl groups in MTX. It was functionalized with thymine—nucleobase forming triple hydrogen bonding with 2,6-diaminopyridine in MTX. Additionally, the NPs were covered with lactobionic acid (LA) to increase targeting abilities. Using two different non-covalent interactions significantly improved drug loading, the nanocarrier was pH sensitive, and LA coating positively affected the increase in cytotoxicity and more effective inhibition of cancer cell growth. A slightly different idea published recently, was to modify chitosan with two components—folic acid and L-cysteine, Figure 7 [67]. FA was responsible for active targeting to folate receptors, while L-cysteine was for redox responsiveness in high glutathione concentrations. The material demonstrated good stability in physiological conditions while quickly releasing MTX in a simulated cancer-reducing environment.

Gemcitabine (GEM) and cytarabine (CYT) are characterized by a similar structure. GEM is an analog of deoxycytidine used to treat various cancers. However, its short half-life in human plasma and high toxicity requires searching for effective delivery systems. Sialic acid-conjugated chitosan NPs were proposed as a targeted medium for GEM delivery to non-small cell lung cancer [68]. On the other hand, intra-vaginal hybrid nanoparticles dedicated to cervical cancer were composed of a lecithin lipid core, mucoadhesive CS shell, and a penetration modifier [69]. In contrast to GEM, cytarabine is mainly used in hematological malignancies. This analog of pyrimidine nucleoside is responsible for DNA polymerase inhibition, which ultimately results in the induction of apoptosis and cell death. Similarly to GEM, it is quickly deactivated due to deamination of the pyrimidine ring; thus, it shows deficient activity against solid tumors. Folate-functionalized CS NPs were proposed as a targeted CYT delivery system for breast cancer cells [70]. The cytotoxicity was investigated in folate positive and negative receptors, and only the former showed an increase in cellular uptake. 

Contrary to the antibiotics group, the presented nanocarrier for antimetabolites contains only free forms of active agents. The selected CS-based NPs are collated in Table 3.

### 2.3. Platinum Complexes

Platinum coordination complexes are sometimes categorized as a subgroup of alkylating agents because their mechanism is also based on directly damaging DNA. Cisplatin, cis-[Pt(NH_3_)_2_Cl_2_], is the first platinum-based drug approved; thus, it is applied to treat various cancers. However, similar to other cytotoxic drugs, it demonstrates strong side effects—especially nephrotoxicity and hepatotoxicity. Therefore, also for this drug, various solutions are proposed to reduce adverse effects and improve therapeutic parameters. Folate-targeted lipid chitosan NPs were studied as effective nanocarriers of cisplatin to ovarian and breast cancer cells [71]. Some chitosan amine groups were initially factionalized with folic acid, while the others were with phospholipids. The presence of lipids increased entrapment efficiency, while FA contributed to higher cellular uptake and better cytotoxicity of the reactive agent. A combination of lipids and chitosan should also result in the effective loading of hydrophilic and lipophilic drugs. Therefore, lipid chitosan NPs were also used to co-deliver cisplatin and curcumin to ovarian cancer cells [72]. Curcumin should reduce the nephrotoxicity of cisplatin and increase antitumor efficacy. The construction of the NPs provided structural integrity by chitosan and no drug leakage due to lipid presence. Another approach assumes linking monoclonal antibodies at the surface of cisplatin-loaded CS NPs. For that purpose, rituximab, a monoclonal antibody that targets CD20, was applied [73]. CD20 is a transmembrane protein, defined as a B cell marker and one of the most common genes in melanoma. The presence of CD20 is identified with an aggressive course of the disease. However, in this case, the results were surprisingly unfavorable. The material not containing rituximab was characterized by more significant cytotoxicity concerning the tested cancer cells. 

Oxaliplatin, a platinum complex with 1,2-diaminocyclohexane, is less toxic than other platinum drugs. However, the effective targeting of cancer cells requires a suitable delivery system. Therefore, simple CS NPs were proposed as a nanocarrier for oxaliplatin for the topical treatment of oral tumors [74]. The penetration efficiency was significantly improved by iontophoresis, resulting in a six-fold increase in the mucosa compared to passive delivery of free-drug.

### 2.4. Microtubule Damaging Agents

**Figure 8:**
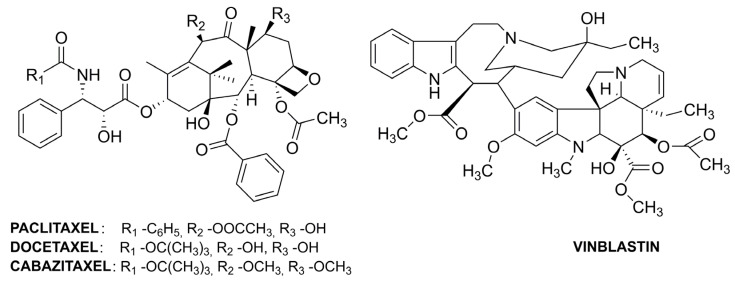
The selected structures of anticancer microtubule inhibitors.

Cellular microtubules are crucial components of the cell cytoskeleton and are formed through the polymerization of tubulin (dimer of α- and β-tubulin). The dynamic equilibrium between microtubules and α-/β-tubulin results in the continuous growing and shortening of the microtubules, stimulating the movement of chromosomes during anaphase. One of the essential functions of microtubules is the segregation of chromosomes during mitosis. The anticancer drugs that are microtubule inhibitors are categorized into taxanes and vinca alkaloids. Both subgroups of antitumor drugs bind to β-tubulin. Taxanes stabilize microtubules; thus, chromosome segregation is disturbed, which causes cell death. The alkaloids prevent β-tubulin polymerization and, thus, destabilize microtubules [75]. The exemplary structures of microtubule damaging agents are presented in Figure 8.

Paclitaxel (PTX) is the most often used taxane drug, but its low therapeutic index and low solubility require the development of systems for its effective delivery. Nanoparticles with three-layers were synthesized using the layer-by-layer method to co-deliver PTX and the antimetabolite drug—5-FU [76]. Initially, chitosan nanoparticles loaded with PTX were prepared and cross-linked with glutaraldehyde. Then, dextran was absorbed on the CS NPs surface by electrostatic effect. Finally, the last layer was composed of 5-FU-loaded chitosan nanoparticles. The presence of several layers decreased the release rate of the reactive agent. The incorporated drugs demonstrated different release mechanisms—for 5-FU, it was diffusion, while for MTX, it was a combination of diffusion and erosion. The cytotoxicity of dual drug-loaded nanocarrier was improved, probably due to better cellular uptake of the NPs. Other multilayer nanoparticles with improved stability in the gastrointestinal tract were proposed for oral delivery of PTX [77]. Initially, quercetin-modified liposomes were used for the entrapment of PTX and then coated with glycocholic acid–chitosan oligosaccharide conjugate. Apical sodium-dependent bile acid transporter (ASBT) is a species expressed on the surface of enterocytes and can be used as a potential target for oral delivery. ASPT affinity to bile acids facilitates crossing the intestinal epithelial barrier by other moieties, thus bile acid-decorated oral drug delivery systems are developed. Bile acids, substrates of ASBT, can be in free form (cholic acids) and conjugated (glycocholic acid) with better hydrophilicity. The inner core of the drug carrier was composed of liposomes decorated with natural quercetin for better encapsulation efficiency and co-delivery of P-gp (transmembrane efflux transporter) inhibitors to overcome one of the biological barriers. A PTX–cholesterol complex was used for encapsulation in glycocholic acid-NPs to increase drug stability. The composite nanocarrier resulted in superior antitumor efficacy of PTX, better bioavailability, and better safety than orally administrated drugs. Other synthesized nanohybrids were designed for the co-delivery of PTX and P-short hairpin (sh)RNA as a combination of chemo- and gene therapy [78]. The shRNA can mediate siRNA, which results in the degradation of targeted RNA. The complex nanocarrier was composed of silica nanoparticles for PTX loading and modified chitosan oligosaccharides for shRNA loading. Cetyltrimethylammonium bromide (CTAB) was used as a template for silica NPs preparation. CS oligosaccharide was modified in several stages. Initially, it was functionalized with 3,3′-dithiodipropionic acid to incorporate a disulfide bond for redox responsiveness and carboxylic groups for further modification (CS-COOH). On the other hand, folic acid was functionalized with polyethylene glycol diamine (FA-PEG-NH_2_). Finally, carboxylic groups in CS-COOH were used to obtain a copolymer of FA-PEG and polyethyleneimine (PEI), FA-PEG-CS-PEI. PEI is often used as a gene carrier. The copolymer surrounded the PTX loaded silica nanoparticles by self-assembly. The complex carrier should demonstrate pH and redox-responsiveness. CTAB is able to form micelles that are destroyed in acidic medium releasing the entrapped PTX. FA acts as a targeting agent that improves drug uptake by breast cancer cells. The reducing tumor environment destroys disulfide bonds, disaggregates low molecular weight polymers and releases P-shRNA.

The nanocarriers for other taxanes were also developed. Docetaxel (DTX) is poorly soluble in water; thus, it requires solubilizers. This disadvantage could be overcome by cholesterol conjugation to chitosan to obtain an amphiphilic CS derivative [79]. The presence of a hydrophobic unit should facilitate the entrapment of a hydrophobic drug. The chitosan–cholesterol conjugate demonstrated enhanced intracellular accumulation and could better overcome multidrug resistance than the DTX solution. On the other hand, dual-ligand targeting by CS NPs was proposed for lung cancer treatment [80]. Folate and epidermal growth factor receptors are overexpressed in lung cancer; thus, folic acid and cetuximab were chosen as their targeting ligands. CS was functionalized with FA, loaded with DTX, and cross-linked with sodium tripolyphosphate (TPP). Then cetuximab, a human chimeric monoclonal antibody that binds to epidermal growth factor receptors, was conjugated to the CS-based nanoparticles. Another approach dedicated to lung cancer treatment was also based on combined chemo- and immunotherapy [81]. This time, a ligand with high affinity to the transferrin receptor overexpressed in many tumors, T7, was chosen for targeted delivery of DTX and curcumin. Another proposal is a combination of chemo- and photodynamic therapy. Cabazitaxel (CTX) and mertansine (MRT), two different microtubule inhibitors—were separately loaded in CS NPs conjugated with the photosensitizer tetraphenylchlorine (TPC) [82]. The aromatic TPC groups were responsible for π-π stacking interactions resulting in high drug loading capacity and strong retention. The cytotoxicity of both drugs incorporated in the NPs was higher than for their free forms.

Vinca alkaloids, similar to taxanes, are responsible for many side effects that stimulate the development of effective delivery systems. Vinblastin (VBL) is an alkaloid drug for different tumor types. Chitosan–hyaluronan NPs were proposed as a nanocarrier for VBL-targeted delivery to CD44-transmembrane receptors overexpressed in tumor cells [83]. Hyaluronic acid (HA) is a polysaccharide actively transported by transmembrane glycoprotein CD44. The nanoparticles were prepared using two techniques: ionotropic gelation of CS with TPP followed by HA coating or direct polyelectrolyte complexation of cationic CS with anionic HA. The latter demonstrated more suitable properties for therapeutic application. The nanocarrier had a solid ability to bind to CD44 transmembrane receptors.

### 2.5. Drugs Affecting the Hormonal Environment

In addition to a large group of cytotoxic drugs, anticancer drugs acting on the hormonal environment are distinguished. Within this group, a fundamental division can be made into steroid hormones (e.g., estrogens) and their antagonists (e.g., tamoxifen among anti-estrogens). The antagonists are steroidal analogs or nonsteroidal compounds that inhibit steroid hormone action [84]. Hormone therapy mainly treats some breast and prostate cancers because their growth depends on sex hormones.

**Figure 9:**
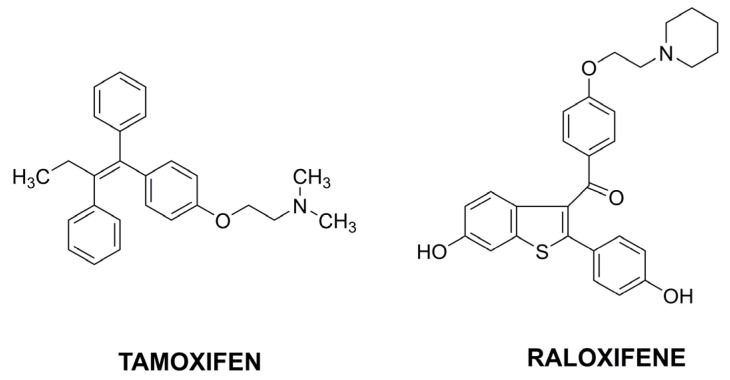
The selected structures of anticancer drugs affecting the hormonal environment.

Among the latest literature reports, tamoxifen (TMX), belonging to the selective estrogen receptor modulators (SERMs), is the most common (Figure 9). TMX is the oldest drug used in hormone-receptor-positive breast cancers. It inhibits the binding of estradiol by estrogen receptors and thus prevents hormone response. It is administrated orally, and its bioavailability is limited due to high metabolism in the liver. Therefore, its entrapment in chitosan nanoparticles could be a solution to improve TMX activity. CS NPs coated with alginate were prepared for the pH-dependent release of TMX [85]. Alginate was applied to protect NPs against the stomach environment and controlled drug release at colon pH. Another approach assumed the coating of TMX-loaded chitosan nanoparticles with hyaluronic acid by forming a covalent bond between the amino groups of CS and the carboxyl groups of HA [86]. HA was chosen as a targeting ligand to membrane glycoprotein CD44. The nanocarrier was applied for targeted TMX delivery against CD44-overexpressing MCF7 and TMX-resistant MCF7 breast cancer cell lines. The drug loaded in the designed nanocarrier demonstrated a high suppressive effect in cancer cells with a lower dosage.

Different hybrid nanoparticles were proposed for co-encapsulation; TMX and curcumin [87]. TMX was entrapped into chitosan, curcumin into lipid, and the resulting mixture formed lipid–chitosan NPs. The presence of two anticancer components should increase the effectiveness in overcoming multidrug resistance. Another nanocarrier proposal concerned another drug from the SERMs group—raloxifene (RLX) [88]. Chitosan nanoparticles were functionalized with cyclo peptide RGD acting as a selective ligand for active targeting to α_v_β_3_ integrins highly expressed in many tumors. The nanoparticles demonstrated higher cellular uptake and cytotoxicity in an acidic tumor environment than in physiological conditions.

Despite an extensive range of drugs used in cancer therapy, other compounds are of little interest in the context of their loading in chitosan-based nanoparticles. Therefore, this study did not refer to individual articles on other drugs. On the other hand, many original papers concern research studies on compounds of natural origin with excellent antitumor activity but not yet approved for anticancer therapy.

### 2.6. Potential Anticancer Agents

**Figure 10:**
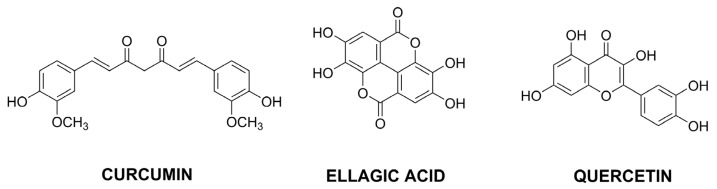
The structures of selected polyphenols with anticancer properties.

Research on new active substances in cancer therapy is an ongoing interest. As in the case of drugs available on the pharmaceutical market, potential new therapeutic agents also often require systems for effective delivery. Particular attention is paid to compounds of natural origin obtained from plants, among which many original articles concern single compounds, mainly from a broad group of polyphenols and essential oils. Different chemical compounds, including flavonoids, stilbenoids, lignans and polyphenolic acids, represent polyphenols (Figure 10). On the other hand, essential oils are complex mixtures containing two general classes of compounds—terpenes and phenylpropanoids. Examples of CS-based nanoparticles used for delivery of potential natural anticancer agents are collated in Table 4.

#### 2.6.1. Encapsulation of Polyphenols

Recently, probably the most favored active compound from the group of polyphenols is curcumin (CUR), extracted from the saffron (*Curcuma longa*) rhizome and used as a spice or yellow colorant of food [26]. In vitro and in vivo studies have demonstrated its anti-inflammatory, antioxidant, antibacterial, antidiabetic, and anticancer activities. The mechanism of antitumor activity is still under discussion, but it probably acts via the induction of cell apoptosis. On the other hand, CUR is very unstable due to its sensitivity to light or temperature and decomposition in neutral pH. It is also poorly soluble in aqueous media and absorbed after oral administration. Therefore, CUR encapsulation in nanocarriers is a reasonable solution to increase biodistribution and bioavailability.

CUR-loaded fungal chitosan NPs were obtained by ionotropic gelation in the presence of TPP and applied to evaluate the anticancer activity against different cancer cells [89]. Such a simple nanoplatform enabled augmenting CUR activity and reduced the viability of cancer cells. On the other hand, a self-assembly technique was used to obtain lecithin–chitosan NPs as a CUR nanocarrier [90]. Electrostatic attraction between a negatively charged phospholipid and a positively charged polysaccharide resulted in a delivery system with improved stability, higher encapsulation rate, improved bioavailability, and effectiveness of the active agent. The stability of curcumin can also be improved by the formation of boronate esters with the phenylboronic acid (PBA) group. Therefore, chitosan oligosaccharides were modified with 3-carboxyphenylboronic acid and then used for CUR loading [91]. A boronate ester between CUR and PBA and hydrogen bonding between CUR and CS resulted in high drug loading. The nanocarrier was sensitive to mildly acidic pH and reactive oxygen species. Another solution for increased CUR stability inside the nanocarrier was based on the inclusion complex formation between the host molecule—cyclodextrin and the guest molecule—curcumin, and their further encapsulation in a chitosan matrix [92]. Cyclodextrins are characterized by lipophilic cavities and hydrophilic outer surfaces; thus, they can be used as a host–guest delivery system for hydrophobic species. The formation of a cyclodextrin complex encapsulated in CS NPs should further improve CUR stability in physiological conditions.

The anticancer properties of CUR could be Increased by its chemical modification with succinic anhydride. Curcumin diethyl dissucinate has better stability in physiological conditions and higher antitumor activity than curcumin, but is also poorly water-soluble. Thus succinylated-CUR was successfully encapsulated in mannose-modified chitosan, as mannose receptors are highly expressed in cancer cells [93]. Another nanocarrier for oral drug administration was composed of chitosan–alginate NPs [94]. The nanoplatform demonstrated good stability in the simulated digestive fluids and ultraviolet light and sustained release of the active component in the digestive and body fluids. Therefore, the bioaccessibility and bioavailability of the drug were significantly increased, resulting in better cellular uptake and cytotoxicity against selected cancer cells.

Several reports also describe more sophisticated systems for curcumin delivery or those dedicated to the co-delivery of several anticancer agents [43,72,87]. Magnetic iron(II, III) oxide nanoparticles were coated with CUR-loaded carboxymethyl CS and combined with hyperthermia as a proposed strategy for breast cancer treatment [95]. Hyperthermia is based on increasing the temperature in the area of cancer cells, which should lead to their death or increase the toxicity of the chemotherapeutic agent. The treatment efficacy was increased compared to free CUR, and the combination of the two methods significantly improved breast cancer cell apoptosis compared to each method alone. On the other hand, a complex nanohybrid with optical properties and pH sensitivity was designed using mesoporous silica, CUR-loaded chitosan, and gold with attached Mucin-1 aptamer as a receptor for MUC-1 positive cell lines in breast and colon cancers [96]. The presence of gold nanoparticles imparts fluorescence emission properties; thus, the nanoplatform could be used not only for CUR delivery but also as a cancer theranostic for pH-dependent fluorescence imaging. The possibility of fluorescence monitoring can be used for tracing targeted CUR delivery.

The literature reports on other potential agents with anti-cancer properties appear less frequently, but it is worth analyzing the proposed solutions for their effective delivery. In addition to curcumin, other flavonoids are also being investigated as anti-cancer agents. Quercetin (QRT) is often studied because of its antioxidant, anti-inflammatory, and antitumor activities [97]. It is sometimes combined with cytotoxic drugs to increase the anticancer effect [53] or sensitize cancer cells resistant to a particular drug. The latter approach was successfully used for paclitaxel-resistant lung cancer cells [98]. The presence of QRT enhanced the cytotoxicity of PTX. Additionally, attached cetuximab was a ligand of epidermal growth factor receptor for better PTX targeted delivery to non-small cell lung cancer. Another flavonoid, hesperetin, can inhibit cell growth in various types of cancer. This property was adapted to obtain chitosan-based NPs targeting colon cancer stem cells [99]. The nanocarrier was composed of folic acid functionalized chitosan with conjugated hesperetin and attached double cortin like kinase 1 antibody. The proposed targeting of cancer stem cells should reduce cancer initiation, progression, and metastasis. Among other active antitumor agents, gallic acid was successfully loaded in carboxymethyl CS NPs to increase its absorption and bioavailability [100], while ellagic acid-loaded CS NPs were coated with tween-80 to increase their therapeutic effect against breast cancer [101].

#### 2.6.2. Encapsulation of Multicomponent Plant Extracts

Essential oils (EOs), extracted from various parts of plants, are characterized by different biological activities, like antioxidant, antibacterial, antifungal, antiviral, and anticancer. However, their high volatility, water insolubility, and high instability require some protection systems to maintain their properties in the biological environment.

*Cynometra cauliflora* EOs, mainly composed of terpenoids, were extracted from the leaf, twig, and fruit of a fruit tree (Malaysia), and after that, nano encapsulated in chitosan by the emulsion-ionic method [102]. The entrapment in the polysaccharide NPs improved Eos’ biological properties, demonstrating antimicrobial activity against diabetic wound microorganisms and cytotoxic activity on the breast MCF-7 cancer cell line. Similarly, *Zataria multiflora* essential oils induced apoptosis, generating reactive oxygen species and damaged DNA in cancer cells after emulsification in CS NPs [103]. The analogous procedure was successfully adapted for the entrapment of EOs extracted from a herb mainly distributed in Turkey and Iran—greater celandine (*Chelidonium majus* L.) [104]. However, the oils were separately extracted from roots and leaves to compare their effectiveness against the MCF-7 cancer cell line. Although the nanoencapsulation of both EOs resulted in significantly higher cytotoxicity against breast cancer cells than their free forms, the highest apoptosis rate was obtained for the ones extracted from leaves.

The same procedure was used to investigate the antitumor effect of three EOs from the citrus family—bitter orange, lemon, and orange against melanoma and breast cancers [105]. The major component of these EOs is limonene, a monoterpene with abilities to inhibit tumor initiation, growth, angiogenesis, and apoptosis of tumor cells. Therefore, comparison studies of anticancer effectiveness between encapsulated limonene and limonene-rich EOs were performed. Essential oils from lemon and orange had much higher limonene content (up to 70%) than bitter orange (about 30%); thus, their anticancer activity after encapsulation in NPs was sufficient to be considered potential green anticancer agents. On the other hand, eugenol-rich EOs were studied as active agents against breast and skin cancer cell lines [106]. The EOs extracted from Syzygium aromaticum contained about 65% of eugenol, a phenylpropene with anti-inflammatory, antioxidant, antimicrobial, and anticancer properties. The antioxidant properties of the EOs were slightly lower than eugenol, while the anticancer effect was significantly improved after nanoencapsulation in chitosan NPs. Therefore, the eugenol-rich EOs could be considered potential chemopreventive agents. Similar assumptions and observations were obtained for cinnamaldehyde-rich EOs extracted from cinnamon and incorporated into CS NPs [107]. Additionally, several reports focused on major components of essential oils as green anticancer agents, including terpineol [108], santalol [109], or thymoquinone [110,111].

A slightly more complex carrier was proposed for jasmine oil loading by crosslinking pectin with calcium ions in the presence of chitosan to obtain pectin/chitosan NPs [112]. The nanoencapsulation resulted in improved thermal stability and antioxidative ability of jasmine oils, while its anticancer properties were 13-fold higher than the pure oil. On the other hand, *Artemis vulgaris* L. EOs were delivered by poly(lactic-co-glycolic acid), PLGA, and NPs covered with folic acid-functionalized chitosan [113]. PLGA is a biodegradable synthetic polymer that effectively protects active agents, but cannot specifically interact with cells or proteins. Therefore, FA-modified chitosan was used as a targeting ligand to folate receptors overexpressed in tumor cells. An analogous nanocarrier was adapted for encapsulation of smoke extract from *Peganum harmala* for targeting breast cancer cells [114]. *P. harmula* seeds were burned, and the smoke components were used as a potential anticancer agent.

Apart from essential oils, aqueous plant extracts are also applied as potential active agents in antitumor therapy. *Epilobium parviflorum* is a powerful medicinal plant rich in tannins, flavonoids, or polyphenolic acids. The leaves were used to prepare aqueous extract and loaded into CS NPs [115]. The extract can interact with DNA by hydrogen bonding; thus, it can be considered a potential drug targeting DNA in cancer treatment. On the other hand, the CS NPs containing rapeseed pollen extract were found to reduce MCF-7 cell viability and increase the expression of genes associated with apoptosis [116]. Additionally, free radical inhibition and anti-inflammatory properties assume they can be used as a chemo-preventing agent in breast cancer treatment.

**Table 4:** Examples of CS-based nanoparticles for effective targeted delivery of potential anticancer agents.

Type of Modification/Functionalization	Loaded Drug	Carrier Advantages	Reference
Phenylboronic acid	CUR	High loading Efficient release	[91]
Succinic anhydride Mannose	CUR	Enhanced antitumor properties Improved targeting	[93]
Iron(II, III) oxide Hyperthermia	CUR	Improved cytotoxicity	[95]
Cetuximab	QRT and PTX	Improved targeting Sensitizing drug-resistant cancer cells to PTX	[98]
Poly(lactic-co-glycolic acid) Folic acid	EOs	Improved stability Enhanced antitumor properties Improved targeting	[113]
Poly(lactic-co-glycolic acid) Folic acid	Smoke extract	Improved stability Enhanced antitumor properties Improved targeting	[114]

## 3. Conclusions

Based on the latest literature reports, it can be seen that the use of unmodified chitosan nanoparticles as carriers of anticancer drugs is increasingly abandoned. It is primarily due to the lack of targeted delivery of active substances to cancer cells and the insufficient stability of chitosan in the digestive tract or circulating blood. The exceptions are essential oils—multicomponent potential active substances, for which bare chitosan nanoparticles as nanocarriers in cancer therapy are a common practice. 

The stability of CS-based NPs in physiological conditions is often improved by covering them with either synthetic (e.g., polyethylene glycol) or natural (e.g., alginate) polymers. Such nanocarriers are usually sensitive to pH and capable of drug release in mildly acidic conditions, characteristic of tumor tissues. Another common practice is incorporating disulfide bonds through modification of chitosan using the polysaccharide amine functional groups. Disulfide bonds are sensitive to reducing environments, i.e., the high glutathione concentration observed in cancer cells. It is therefore applied to destroy the carrier and release the active substance in the targeted environment of tumor cells. Both treatments protect healthy cells from the toxic side effects of the active substances and increase their concentration in the desired environment, which should consequently lead to a reduction in their doses compared to free drug administration.

However, the above treatments still seem insufficient; hence chitosan is often modified with ligands that ensure active targeting of the carrier to the region with overexpression of given receptors. Folic acid is probably the most commonly used compound for this purpose, as folic receptors are highly expressed in various types of cancer. A beneficial effect manifested by an increase in the cytotoxicity of active substances is observed for systems demonstrating pH and/or redox sensitivity combined with targeted ligands. At the same time, such modifications insignificantly increase the cost of carriers, which is essential from a practical point of view and their chances of practical use.

Another solution is based on loading several substances with anticancer activity in the carrier. For this purpose, a combination of various cytotoxic drugs is used, as well as the addition of anti-inflammatory drugs and substances of natural origin with a broad spectrum of properties, including anticancer. This procedure allows for a synergistic effect and effective treatment of cancers resistant to certain drugs. Therefore, significant attention is paid to compounds of natural origin (e.g., curcumin) as potential agents that could be used in green cancer therapy. As this group of compounds is susceptible to biological conditions, intensive research is also underway on effective carriers that deliver them to the desired area.

On the other hand, a vast number of original scientific papers from the last three years do not specify the optimal parameters of the carrier for a given drug. All conducted treatments concern increasing the stability of the carrier in physiological conditions and thus protecting the drug, as well as increasing the targeted delivery and release of the drug in cancer cells. The investigated parameters are usually referred to the free form of the drug. However, despite often auspicious data, there is no information on how a given carrier would perform its functions using a different active substance. Therefore, the fundamental question arises whether a given carrier would allow obtaining analogous parameters significant in anti-cancer therapy using an active substance with a similar chemical structure or even representing a different group of compounds. For this reason, continued research in this area is necessary. In the author’s opinion, the proposed methods of protecting drugs and increasing their concentration in the targeted environment are effective and are an excellent alternative to traditionally administered drugs. At the same time, research should be undertaken on a given carrier based on chitosan nanoparticles using various model anticancer drugs. Thus, a universal medium dedicated to the same class of compounds could be obtained.

## Figures and Tables

**Table 1:** The number of original articles on chitosan nanoparticles, including drug delivery systems and selected anticancer drugs, based on data from the Scopus database (15 December 2022) for 2020–2022.

Selected Terms *	2020	2021	2022
Chitosan nanoparticle(s), CS NP(s)	1963	2159	2263
CS NPs and drug delivery system(s)/targeted drug delivery	503	504	522
CS NP(s) AND cancer	291	347	333
CS NP(s) AND cisplatin	14	22	16
CS NP(s) AND doxorubicin	87	87	73
CS NP(s) AND methotrexate	12	14	15
CS NP(s) AND paclitaxel	28	26	15
CS NP(s) AND 5-fluorouracil	23	19	26
CS NP(s) AND curcumin	72	80	86

* terms of the article title, keywords, or abstract.

**Table 2:** Examples of CS-based nanoparticles for effective targeted delivery of doxorubicin.

Type of Modification/Functionalization	Form of Loaded DOX	Carrier Advantages	Reference
Polyethylene glycol Monoclonal antibodies	Free	High selectivity High cytotoxicity against breast cancer cells	[29]
Polyethylene glycol High-intensity focused ultrasound	Free	Deep tumor penetration	[30]
Pluronic derivative	Free	Better drug concentration in tumor cells Higher oxidative stress	[34]
Oligosaccharides combined with benzaldehyde-terminated PEG	Imine bonds with benzaldehyde	pH sensitivity due to aniline bond destruction in the tumor environment	[36]
Magnetic core Folic acid	Free	External magnet support Targeted folate receptors	[38]
Catechol Hyaluronic acid	Free	High mucoadhesive ability Local drug administration	[39]
Benzylguanidine Lactobionic acid	Free	Targeted delivery to CXCR 4 positive tumors	[40]
Folic acid 2-(Diisopropyloamino) methacrylate	Free with co-loaded survivin	Combined gene- and chemotherapy Targeted folate receptors	[43]
Raloxifene	Free	combined therapy with selective estrogen receptor modulator	[44]
Estrone	Conjugate with estrone	Dual-targeted estrogen receptors	[45]
Glycol Hyaluronic acid	Attached to CS with co-loaded celecoxib	Synergism of active agents pH-sensitive drug attachment	[48]
Stearic acid	Free with co-loaded curcumin	Redox-sensitive disulfide bond Encapsulation of hydrophilic and hydrophobic drugs Synergism of active agents	[50]
Cinnamaldehyde Lactobionic acid	Free	Synergism of active agents Better antitumor effect Lower toxicity to healthy cells	[55]

**Table 3:** Examples of CS-based nanoparticles for effective targeted delivery of antimetabolites.

Type of Modification/Functionalization	Loaded Drug	Carrier Advantages	Reference
Polycaprolactone	5-FU	Higher antineoplastic activity in head and neck tumors	[57]
Folic acid Calcium alginate	5-FU	Targeted folate receptors Stability in the gastric environment Mucoadhesive properties	[58]
Cerium oxide	5-FU	The higher scavenging ability of reactive oxygen species	[61]
Polypyrrole Carboxymethyl cellulose	5-FU	Synergistic chemo and photothermal therapy	[64]
Thymine Lactobionic acid	MTX	Improved drug uptake Improved targeting	[66]
L-cysteine Folic acid	MTX	Improved targeting	[67]
Lecithin	GEM	Improved vaginal penetration	[69]
Folic acid	CYT	Better targeting for folate-positive breast cancer cells	[70]

## Data Availability

Not applicable.
